# Patient delay and its clinical significance among head and neck cancer patients in Hungary

**DOI:** 10.3389/pore.2023.1611206

**Published:** 2023-08-22

**Authors:** Kornél Dános, Angéla Horváth, Judit Halász, László Tamás, Gábor Polony

**Affiliations:** ^1^ Department of Otorhinolaryngology, Head and Neck Surgery, Faculty of Medicine, Semmelweis University, Budapest, Hungary; ^2^ Department of Pathology, Forensic and Insurance Medicine, Faculty of Medicine, Semmelweis University, Budapest, Hungary

**Keywords:** survival, diagnosis, head and neck cancer, patient delay, symptom

## Abstract

**Introduction:** Head and neck cancers represent a major health problem in Hungary. With their high incidence and mortality rates, Hungary is one of the world leaders in these indicators. The length of patient delay, defined as time from onset of symptoms to first medical consultation, is unknown in Hungarian patients with head and neck cancer. We aimed to use a representative sample of the Hungarian head and neck cancer patient population to determine patient delay according to disease localization and stage and to identify correlations with other clinical parameters.

**Methods:** In our retrospective study, we reviewed patient documentation. For the inclusion, the patients had to be diagnosed with malignant tumors of the oral cavity, oropharynx, hypopharynx or larynx at the Department Head and Neck Surgery of Semmelweis University between 2012 and 2017.

**Results:** We identified 236 patients who met the inclusion criteria. The median delay was 9.5 weeks (range 0–209 weeks) and the mean delay of patients was 17.57 weeks (SD 23.67). There was a significant difference in patient delay data by location. Among glottic cancers, the most common diagnosis was an early stage (67%), compared with other localizations, including most commonly the oropharynx (81%) and hypopharynx (80%), where a locoregionally advanced stage was more frequent.

**Discussion:** Compared to data from different countries, the delay of Hungarian patients with head and neck cancer is significantly longer, which may contribute to the high mortality in Hungary. Screening and patient education in high-risk groups could contribute to earlier diagnosis and thus improve prognosis.

## Introduction

Hungary has the third highest incidence of head and neck squamous cell cancer (HNSCC) and the second worst cancer-related mortality in the world, causing an important health problem [1].

There are many articles in the international literature on patient delay as an important factor in late diagnosis, but no data are available for Hungary [2].

Diagnostic delay refers to the total period of time from the onset of symptoms to (histological and radiological) diagnosis. Diagnostic delay is generally separated into two phases: patient delay defined as the time period from the beginning of symptoms to the point where the patient seeks medical care and system delay is the elapsed time between first contact with a healthcare professional and the definitive diagnosis/treatment initiation [3].

In the case of suspected cancer, system delay can be shortened by accurate patient pathways, as such the “14-day rule,” established by the Hungarian Government in 2014, aiming to reduce waiting times for staging-purpose imaging tests, and the 21 (30)-day rule which aims to shorten the time in which patients are referred to multidisciplinary tumor boards. Nevertheless, it is known that the length of time a patient waits from the first presentation of his/her first symptoms is crucial in treatment success, head and neck cancer patients very often seek medical attention for life-threatening symptoms only. It is also known that patient delay is multifactorial, including socioeconomic conditions, marital status, psychopathology, denial, and is therefore difficult to influence the time to first medical consultation [4]. No high-level evidence is available for HNSCC screening in the general population. Screening of patients with risk factors would be most recommended, but this patient group is difficult to access to make them adopt this awareness [5]. This underlines the importance of activities such as the Make Sense campaign launched by the European Head and Neck Cancer Society in 2013 to raise awareness of head and neck cancer [6].

The present study aimed to evaluate the patient delay in this specific and special Hungarian (based on incidence and mortality) patient population, and its correlations with clinical parameters.

## Patients and methods

We aimed to enroll all patients in this retrospective, single-institution study after being diagnosed (histologically) with squamous cell cancer of the oral cavity, oropharynx, hypopharynx, or larynx between September 2012 and August 2017 at the Department of Otolaryngology and Head-Neck Surgery, Semmelweis University, Hungary.

The main exclusion criteria were: patients who were not diagnosed (by biopsy) at our tertiary referral center or had primaries in other head and neck sites (e.g., nasopharynx, nasal cavity, paranasal sinuses, salivary gland, or skin, furthermore those with unknown primaries) or had previous treatment for head and neck tumor or/and underwent oncological treatment in the last 5 years. Patients (22 cases) who had been diagnosed in other hospitals were excluded.

In our study, we evaluated retrospectively the patients’ medical records (discharge summaries, multidisciplinary tumor board reports, histological reports, and imaging studies).

The TNM stages were defined using the seventh edition of the UICC TNM system [7].

To determine the HPV status in oropharyngeal cancers, as in the clinical practice and current guidelines, we used immunohistochemical (IHC) detection of the tumor suppressor p16^INK4^ (Vitro Master Diagnostics, mouse anti-human p16-INK4A monoclonal antibody (MX007), 1:100). We used the primary antibody at 37°C with an incubation time of 10 min, at least 70% combined nuclear and cytoplasmic staining of the tumor tissue was necessary for positivity. This method is a reliable, surrogate marker for testing human papillomavirus (HPV) in oropharyngeal carcinoma [8].

For survival analyses, disease-specific survival was used: the statistical event was defined as death due to cancer or the presence of a tumor at the last check-up, while tumor-free control or death due to other diseases was not defined as an event.

Statistical analysis of the data—with descriptive statistics and correlation analyses—was performed using the Kruskal-Wallis test, Mann-Whitney U test, Chi-square test (Fisher’s exact test), Kaplan-Meier estimates with log-rank tests.

We used SPSS Statistics for Mac v.28.0 (IBM Corp., Armonk, NY) for the analyses.

Patient delays were reported using the median values. We considered *p* < 0.05 as statistically significant.

Our research was carried out with the ethical permission of Semmelweis University (SE IKEB 105/2014).

## Results

236 patients were identified meeting the criteria. We examined 198 men and 38 women. The mean age of the patients at the time of diagnosis was 61 years (40–92 years). The patient data is presented in Table 1. The median delay was 9.5 weeks (range 0–209 weeks), mean patient delay was 17.57 weeks (Std. deviation 23.67).

**TABLE 1:** Descriptive statistics of patients involved in our study.

Clinicopathological features	All patients (*N* = 236)
Gender	
Male	198 (84%)
Female	38 (16%)
Alcohol consumption	
Never	59 (25%)
Previously	25 (10%)
Currently	105 (45%)
Not available	47 (20%)
Smoking	
Never	11 (4.7%)
Previously	46 (19.5%)
Currently	163 (69%)
Not available	16 (6.8%)
Age	61 years (40–92 years)
Stage	
T	
1	36 (15.2%)
2	53 (22.5%)
3	52 (22%)
4a	55 (23.4%)
4b	26 (11%)
Not available	14 (5.9%)
N	
N0	112 (47.4%)
N+	110 (46.6%)
Not available	14 (5.9%)
M	
M0	223 (94.5%)
M1	13 (5.5%)
Not available	0
Grade	
1	13 (5.5%)
2	66 (28%)
3	53 (22.5%)
Not available	104 (44%)
Sites	
Oral cavity	14 (5.93%)
Oropharynx p16 positive	28 (11.87%)
Oropharynx p16 negative	18 (7.63%)
Oropharynx p16 not available	29 (12.29%)
Hypopharynx	51 (21.62%)
Supraglottic larynx	24 (10.17%)
Glottic larynx	60 (25.42%)
Transglottic larynx	10 (4.23%)
Subglottic larynx	1 (0.42%)
Not assessable	1 (0.42%)

As for the tumor localizations in Table 2, the median delay was the longest in patients with supraglottic cancer, followed by glottic and transglottic tumors, while the patient delay was 8 weeks for oral and oropharyngeal cancers (*p* = 0.004).

**TABLE 2:** Delay in weeks according to the region (*p* = 0.004).

Localization	Number	Mean patient delay (weeks)	Median patient delay (weeks)
Oral cavity	14	17.09	8
Oropharynx	75	11.18	8
Hypopharynx	51	13.9	11
Supraglottic larynx	24	27.75	*17.5*
Glottic larynx	60	25.9	15
Transglottic larynx	10	16.33	13
Subglottic larynx	1	Value = 4	

Regarding the gender distribution of patient delay: we did not find a significant difference between the median patient delays by gender: men’s median patient delay was 9 weeks, while it was 10 weeks among women (*p* = 0.237). In the women group, there was one remarkable outlier, who waited 209 weeks with her symptom, explaining the difference in the mean value (Table 3).

**TABLE 3:** Delay in weeks according to the gender.

Gender	Percent (%)	Mean patient delay	Median patient delay
Women	16.1	26.58 (1–209)	10
Men	83.9	15.79 (0–107)	9

An overview of the symptoms by region shows the following (Table 4): in case of oral cancers, difficulty in swallowing (42.9%) was the most frequent symptom, 28.6% of this patient group had a sore throat and 14.3% reported weight loss, as significantly more common complaints than in other regions. We found that dysphagia was common among oropharyngeal cancer patients (40%), however, throat pain (48%) was even more frequent and neck mass was significantly more common than in other regions (32%).

**TABLE 4:** Symptoms (in percentage) with significance value and median patient delay (in weeks) according to the region.

Regions Complaints	Oral cavity	Oropharynx	Hypopharynx	Supraglottic larynx	Glottic larynx	Transglottic larynx	Subglottic larynx	*p*
Sore throat	28.6	*48.0*	25.5	29.2	3.3	0	0	<0.001
Hoarseness	0	4.0	21.6	29.2	*96.7*	60.0	*100*	<0.001
Neck mass	14.3	*32.0*	27.5	20.8	0	10.0	0	<0.001
Difficulty in swallowing	42.9	40.0	*47.1*	25.0	0	20.0	0	<0.001
Weight loss	*14.3*	4.0	9.8	4.2	0	0	0	0.149
Earache	7.1	13.3	*19.6*	4.2	0	10.0	0	0.027
Hemoptysis	0	0	2.0	4.2	0	0	0	0.513
Trismus	0	2.7	0	0	0	0	0	0.636
Dyspnea	0	5.3	5.9	4.2	3.3	20.0	100	<0.001
Median patient delay in weeks	8.0	8.0	11.0	17.5	15.0	13.0	4.0	

Of all the regions, dysphagia was the most common among patients with hypopharyngeal cancer (47.1%), which was also found to be significant, but sore throat (25.5%), hoarseness (21.6%), neck mass (27.5%) and earache (19.6%) were also common symptoms in this group, of which earache showed to be significant, compared to other regions. In case of supraglottic tumors, the symptoms showed higher variability such as sore throat (29.2%), hoarseness (29.2%), dyspnea (25%), and neck mass (20.8%).

Patients with glottic and transglottic carcinoma presented significantly more often with hoarseness. The only subglottic cancer patient had hoarseness and dyspnea.

It is important to see what the median patient delay time was per region in light of the characteristic complaints.

Patients with a main complaint of hoarseness or difficulty in swallowing had significantly higher waiting times, compared to those without either of these two symptoms (*p* < 0.001 and *p* = 0.022, respectively). There were no differences in waiting times for sore throat, neck lump or earache. Due to the small number of patients, correlation analysis could not be performed for hemoptysis and trismus (Table 5).

**TABLE 5:** Patients’ delay time according to complaint in weeks *p < 0.05*.

Complaints	Median patient delay in weeks		*p*
	With symptom	Without symptom	
Sore throat	10	9	0.985
Hoarseness	15	8	*<0.001*
Neck mass	8	10	0.215
Difficulty in swallowing	8	11.5	*0.022*
Weight loss	7	10	—
Earache	8	10	0.140
Hemoptysis	0	10	—
Trismus	32	9.5	—
Dyspnea	6.5	10	—

The clinical stage at the time of the diagnosis is of utmost importance regarding the prognosis of head and neck cancers [9]. In our data, 67% of glottic cancers were diagnosed in the early stage, while the tumors in other localizations were diagnosed predominantly in a locoregionally advanced stage. In oropharyngeal and hypopharyngeal cancers, distant metastasis at the time of diagnosis occurred in 10% (Table 6).

**TABLE 6:** Cancer stage according to the cancer site: *p < 0.05*.

	Oral cavity	Oropharynx	Hypopharynx	Supraglottic larynx	Glottic larynx	Transglottic larynx
Early stage (T1-2N0M0)	3	7	5	8	*38*	3
	23%	10%	10%	36%	*67%*	30%
Locoregionally advanced stage (T3-4N0M0, T1-4N1-3M0)	10	*55*	*40*	13	18	7
	77%	*81%*	*80%*	59%	32%	70%
Distant metastasis (T1-4N1-3M1)	0	*6*	*5*	1	1	0
	0%	*10%*	*10%*	5%	2%	0%

**FIGURE 1:**
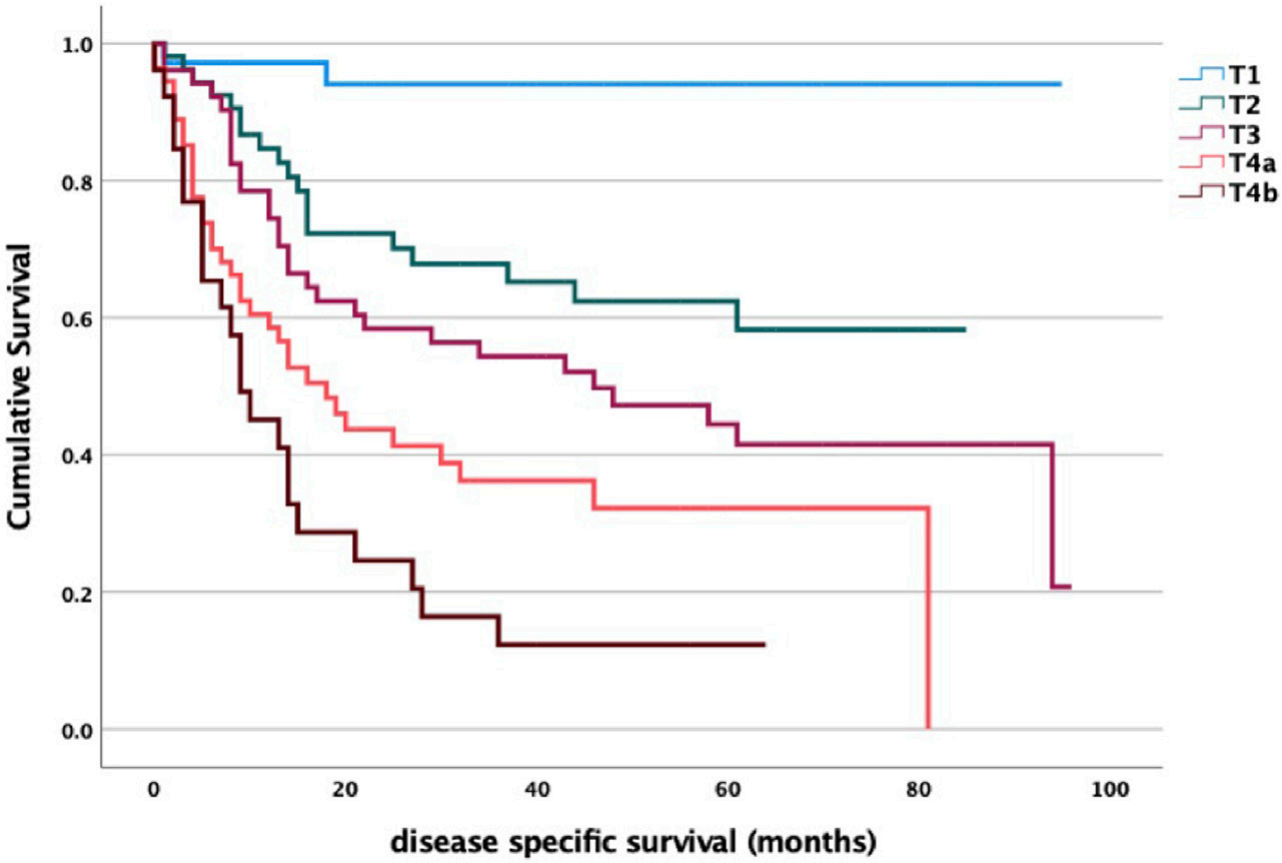
T stage and survival analysis log-rank *p* < 0.001. T1 vs. T2 *p* = 0.025; T1 vs. T3 *p* < 0.001; T1 vs. T4a *p* = 0.003; T1 vs. T4b *p* < 0.001; T2 vs. T3 *p* = 0.099; T2 vs. T4a *p* = 0.105; T2 vs. T4b *p* = 0.005; T3 vs. T4a *p* = 0.805; T3 vs. T4b *p* = 0.108; T4a vs. T4b *p* = 0.316.

**FIGURE 2:**
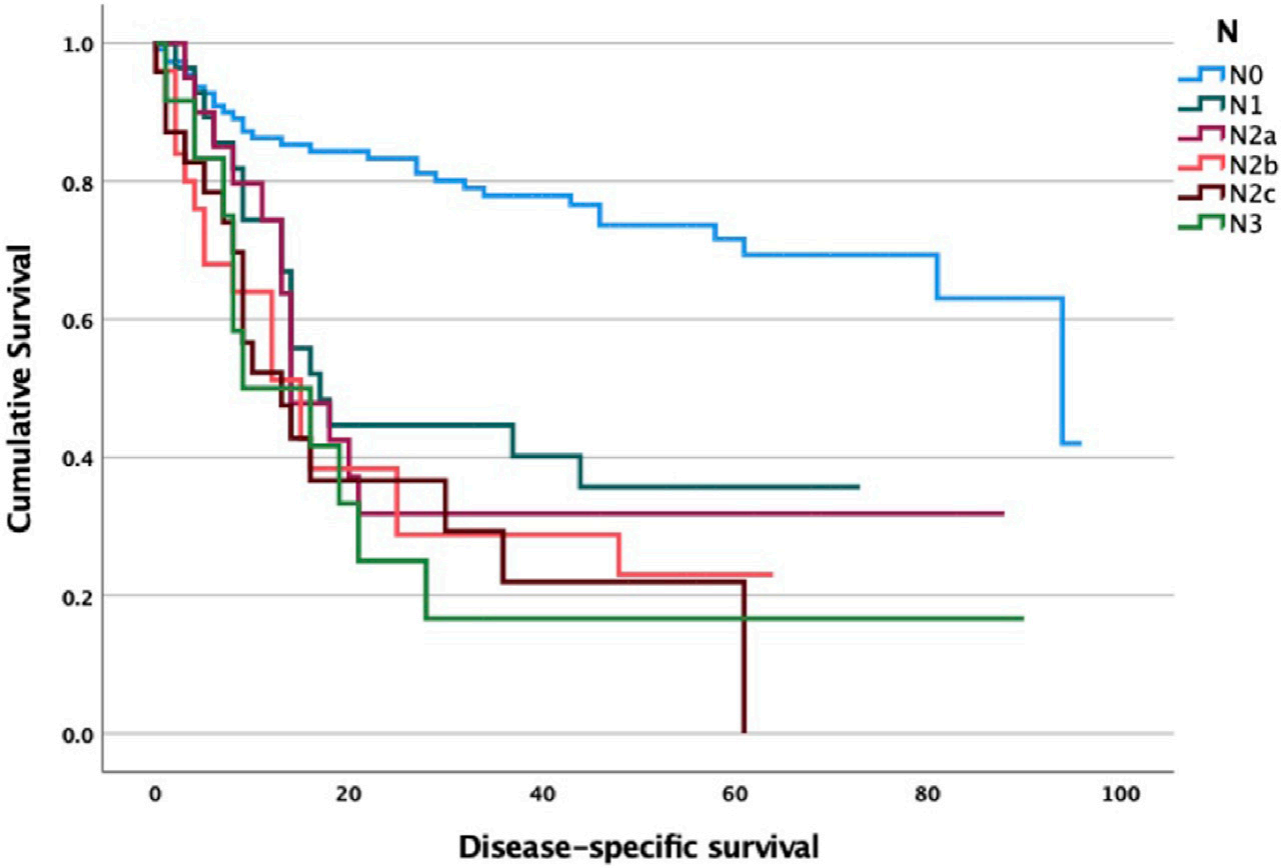
N stage and survival analysis log-rank *p* < 0.001. N0 vs. N1 *p* < 0.001; N0 vs. N2a *p* = 0.005; N0 vs. N2b *p* < 0.001; N0 vs. N2c *p* < 0.001; N0 vs. N3c *p* = 0.049; N1 vs. N2a *p* = 0.957; N1 vs. N2b *p* = 0.433; N1 vs. N2c *p* = 0.103; N1 vs. N3 *p* = 0.791.

**FIGURE 3:**
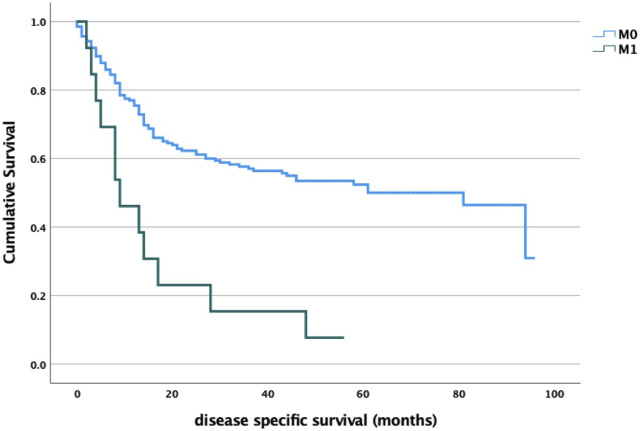
M stage and survival analysis log-rank *p* < 0.001.

Regarding survival data, we found a significant association between stage and disease-specific survival for both T, N and M status (Figures 1–3). Of particular note is the N status, where the localization and number of neck metastases do not influence survival as much as the presence or absence of cervical lymph node metastases at all (Figure 2). In cases of distant metastases, survival was significantly worse (Figure 3).

**FIGURE 4:**
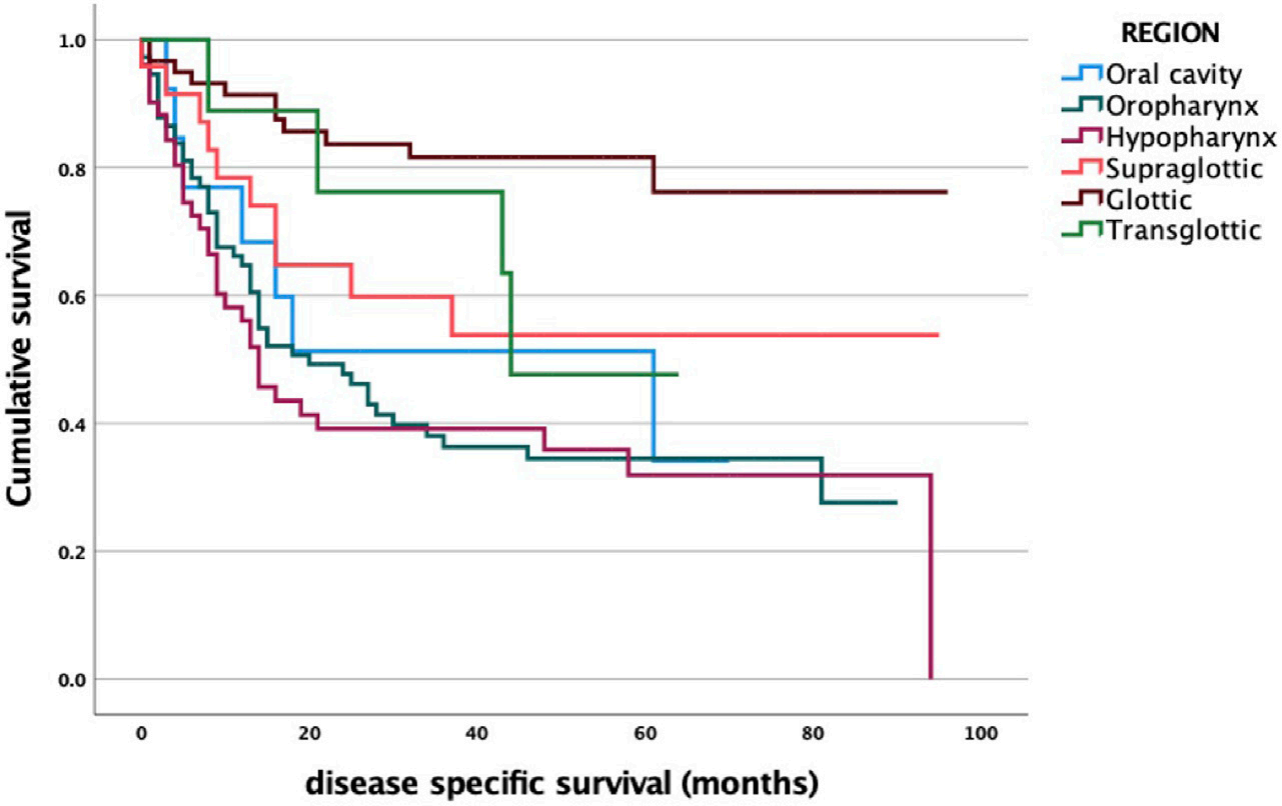
Primary tumor site and survival analysis log-rank *p* < 0.001. oral vs. orophar. *p* = 0.906; oral vs. hypophar. *p* = 0.996; oral vs. supraglot. *p* = 0.860; oral vs. glot. *p* = 0.013; oral vs. transglot. *p* = 0.185; orophar. vs. hypophar. *p* = 0.985; orophar. vs. supraglot. *p* = 0.723; orophar. vs. glot. *p* < 0.001; orophar. vs. transglot. *p* = 0.144; hypophar. vs. supraglot. *p* = 0.603; hypophar. vs. glot. *p* < 0.001; hypophar. vs. transglot. *p* = 0.139; supraglot. vs. glot. *p* = 0.012; supraglot. vs. transglot. *p* = 0.215; glot. vs. transglot. *p* = 0.986.

A significant correlation between tumor localization and survival was found. It is noteworthy that laryngeal cancers (with longer patient delay) have better survival rates compared to the oral cavity and pharyngeal cancers. Based on our previous data, it is remarkable that while the initial stage certainly predicts a high survival rate, in contrast, we found no significant direct association between the length of patient delay and survival (Figure 4).

**FIGURE 5:**
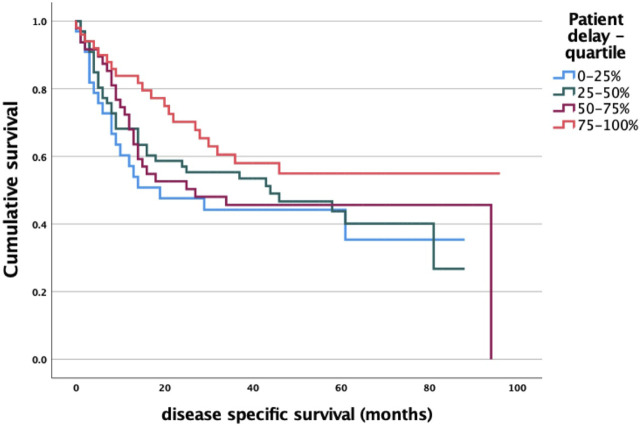
Patient delay and survival analysis log-rank *p* = 0.214.

When correlating quartiles of patient delay time (longest to shortest time interval in quartiles) to survival, we also found that patient waiting time did not significantly affect survival *p* = 0.214 (Figure 5).

We performed a multivariate regression analysis showing that while the prognostic role of stage remained significant, the patient delay was not a significant predictor of survival (Table 7).

**TABLE 7:** Multivariate regression analysis (Cox regression).

	*p*	HR	95% confidence interval (CI)	
			Lower	Upper
Stage	<0.001	5.075	2.756	9.347
Patient delay	0.265	0.990	0.974	1.007

### Role of p16 status in oropharyngeal cancers

As for tumor biology, two groups of oropharyngeal cancers can be distinguished: here we present an overview of the symptoms according to the p16 status. In p16-positive oropharyngeal cancer patients, neck mass as presenting symptom was significantly more common than in p16-negative tumor cases (*p* = 0.003). There was no significant correlation between the patient delay and HPV-positivity status in the oropharyngeal cancer group (*p* = 0.524) (Table 8).

**TABLE 8:** Oropharyngeal cancer symptoms according to p16 status.

	Presence of symptoms in percent		*p*
	p16 positive OPC	p16 negative OPC	
Sore throat	38.9	64.3	0.091
Hoarseness	5.6	3.6	1.0
Neck mass	61.1	17.9	*0.003*
Difficulty in swallowing	16.7	42.9	0.064
Weight loss	0	7.1	0.513
Earache	0	17.9	0.140
Hemoptysis	—	—	—
Trismus	5.6	3.6	1.0
Dyspnea	0	14.3	0.144
Median patient delay in weeks	8 weeks	8 weeks	0.524

The p16 immunohistochemistry was available in forty-six out of seventy-six oropharyngeal cancer patients. Mean patient delay was more for p16 positive patients 14.47 weeks *versus* 9 weeks, while median delay did not differ: 8 weeks for both groups *p* = 0.524 (Table 9).

**TABLE 9:** p16-positivity and patient delay in weeks in oropharyngeal cancers *p* = 0.524.

p16 status	Numbers (available patient delay scores)	Mean delay	Median delay
Positive	18 (17)	14.47 (2–56) weeks	8.00 weeks
Negative	28 (26)	9.81 (2–42) weeks	8.00 weeks
Not available	29		

**FIGURE 6:**
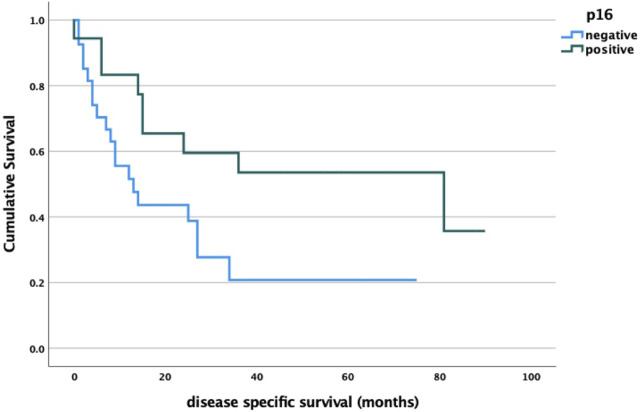
p16 status and survival analysis log rank *p* = 0.032.

As expected, we found a significant correlation between HPV status and survival: p16-negative oropharyngeal cancers were associated with significantly worse survival (Figure 6).

## Discussion

Head and neck cancers can cause a diversity of symptoms. These can be further classified into general symptoms, such as fatigue, weight loss, or site-specific ones, e.g., hoarseness, and sore throat.

Hoarseness can be due to the impaired function and structure of the cover-body system of the vocal cords, vocal fold movement problems associated with direct tumor spread to the paraglottic space or the thyroarytenoid joint, or infiltration of the recurrent laryngeal nerve [10]. Altered voice quality can also occur by structural abnormalities (i.e., presence of the tumor mass) in the pharynx and oral cavity. In our data set, hoarseness was significantly more common in cancers arising in the glottic and subglottic larynx, interestingly, the symptom was also significantly associated with longer patient delay. The latter might be associated with the better biological behavior of glottic cancers [11].

Referred ear pain can occur due to the involvement of the 5th (mainly in oral cancers), more commonly by the 9th and 10th cranial nerves [12]. We found a significant association between otalgia and tumor localization: hypopharyngeal cancers caused ear pain the most frequently.

Sore throat and neck mass, as presenting symptoms were significantly the most common in oropharyngeal cancers, and further analyzed by p16-status, HPV-positive cancer patients presented 3 times more common with neck lump as an initial sign of disease, which phenomenon is due to the often rapidly growing, cystic neck metastases of HPV-related head and neck cancers [13].

Regarding patient delay, when comparing the results of our study on a representative patient population in Hungary (17.57 weeks) with international data (Table 10), similar times were found (17.09 weeks) to the Canadian data [14]. However, the data from Brazil (43.48 weeks, median) were longer than our data [15]. This indicator was lower in the United Kingdom, where the mean patient delay for oral cancer patients was 7.4 weeks [16], another localization of head and neck cancer was given as 12.3 weeks. In South India the median delay was only 4.29 weeks [17]. In the United States, from 1993 to 2004, this result (12.67 weeks) was less than in Hungary, but from 2005 to 2013 it was close to our rate (17.03 weeks) [18]. One-sample test was used to compare whether the difference in the delay times was significant. We found a significant difference between our data and the Brazilian and South Indian results.

**TABLE 10:** Patient delay times by country.

Country	Patient delay (weeks)	Period	Region	*p*-value
Our data	17.57 (mean)	2012–2017		
Canadian [14]	17.09 (mean)	2017–2018	All HNC	0.598
Brazil [15]	43.48 (median)	2011–2012	All HNC	*<0.001*
United Kingdom [16]	7.4 (mean)	1995–1996	Oral	0.307
	12.3 (mean)	1995–1996	All HNC ex. oral	*0.002*
South India [17]	4.29 (median)	2016–2017	All HNC	*<0.001*
United States [18]	12.67 (not reported)	1993–2004	All HNC	*0.001*
	17.03 (not reported)	2005–2013	All HNC	0.573
Iran [20]	23.05 (mean)	2004–2006	All HNC	*0.002*

In reviewing the factors that determine patient survival, one of the first to be mentioned is the size of the primary tumor, the presence of lymph node metastases and distant metastases. The TNM stage is a reliable predictor of survival, which increases with time, and thus with delay time. In advanced cancers, more drastic and radical surgery may be performed, and often the general condition of the patient, which also decreases with time, does not allow curative surgery. In advanced cancers, the side effects of chemo-radiation are also more pronounced. Delay time therefore indirectly determines patient survival [19].

Looking further at the components of survival, it is noted that some localized tumors have been found to have better survival rates despite longer waiting times. These were glottic and p16-positive oropharyngeal cancers. This suggests that the variable biological behavior of tumors with different localization is an important factor for patient survival. Glottic cancers grow more slowly and, due to their location, show obvious symptoms (hoarseness) even when small, which may trigger the patient to seek medical attention. However, our results show that they wait for a long time, which may be because their hoarseness does not cross their stimulus threshold, they probably think it is just a normal part of smoking. Furthermore, due to their lymphatic drainage, glottic tumors tend to metastasize only at an advanced stage.

The better survival of p16 oropharyngeal cancers can be explained by two factors: they have different tumor biology, which makes them more responsive to chemo-radiation, and they are younger, in a better general condition, with less smoking and alcohol-related diseases, so they can tolerate treatment better.

Tumors with different localizations and behaviors overlap in terms of symptoms, so this does not allow us to screen out patients with a more favorable biological behavior being likely to have a good chance in case of a delayed diagnosis. Instead of population-wide screening, the solution would be to screen patients with risk factors—smoking and drinking—so that tumors can be diagnosed and treated at an earlier stage.

A possible weakness of our study is the subjective matter of the patients’ self reported date of symptom onset, however, there is no other option to assess the symptom onset which would be more accurate.

## Data Availability

The raw data supporting the conclusion of this article will be made available by the authors, without undue reservation.

## References

[1] International Agency for Research on Cancer. Globocan: Global cancer observatory (2020). Retrieved from: https://gco.iarc.fr/today/online-analysis-multibars?v=2020&mode=population&mode_population=countries&population=900&populations=900&key=crude_rate&sex=0&cancer=39&type=0&statistic=5&prevalence=0&population_group=0&ages_group%5B%5D=0&ages_group%5B%5D=17&nb_items=10&group_cancer=0&include_nmsc=0&include_nmsc_other=1&type_multiple=%257B%2522inc%2522%253Afalse%252C%2522mort%2522%253Atrue%252C%2522prev%2522%253Afalse%257D&orientation=horizontal&type_sort=1&type_nb_items=%257B%2522top%2522%253Atrue%252C%2522bottom%2522%253Afalse%257D#collapse-others (Accessed July 5, 2023).

[2] Neal RD. Do diagnostic delays in cancer matter? Br J Cancer (2009) 101(2):S9–S12. 10.1038/sj.bjc.6605384 19956171 PMC2790696

[3] Goy J Hall SF Feldman-Stewart D Groome PA. Diagnostic delay and disease stage in head and neck cancer: A systematic review. Laryngoscope (2009) 119(5):889–98. 10.1002/lary.20185 19301409

[4] Rozniatowski O Reich M Mallet Y Penel N Fournier C Lefebvre JL. Psychosocial factors involved in delayed consultation by patients with head and neck cancer. Head Neck (2005) 27(4):274–80. 10.1002/hed.20157 15712292

[5] Franceschi S Barzan L Talamini R. Screening for cancer of the head and neck: If not now, when? Oral Oncol (1997) 33(5):313–6. 10.1016/s1368-8375(97)00034-1 9415328

[6] Reich M Licitra L Vermorken JB Bernier J Parmar S Golusinski W Best practice guidelines in the psychosocial management of HPV-related head and neck cancer: Recommendations from the European head and neck cancer society's make Sense campaign. Ann Oncol (2016) 27(10):1848–54. 10.1093/annonc/mdw272 27456297

[7] Edge SB Compton CC. The American joint committee on cancer: The 7th edition of the AJCC cancer staging manual and the future of TNM. Ann Surg Oncol (2010) 17(6):1471–4. 10.1245/s10434-010-0985-4 20180029

[8] Dok R Nuyts S. HPV positive head and neck cancers: Molecular pathogenesis and evolving treatment strategies. Cancers (Basel) (2016) 8(4):41. 10.3390/cancers8040041 27043631 PMC4846850

[9] Teppo H Alho OP. Relative importance of diagnostic delays in different head and neck cancers. Clin Otolaryngol (2008) 33(4):325–30. 10.1111/j.1749-4486.2008.01704.x 18983341

[10] Ambrosch P Fazel A. Functional organ preservation in laryngeal and hypopharyngeal cancer. GMS Curr Top Otorhinolaryngol Head Neck Surg (2011) 90(1):Doc02–109. Laryngorhinootologie. 10.3205/cto000075 PMC3341579 22558052

[11] Szentkúti G Dános K Brauswetter D Kiszner G Krenács T Csákó L Correlations between prognosis and regional biomarker profiles in head and neck squamous cell carcinomas. Pathol Oncol Res (2015) 21(3):643–50. 10.1007/s12253-014-9869-4 25547827

[12] Chen RC Khorsandi AS Shatzkes DR Holliday RA. The radiology of referred otalgia. AJNR Am J Neuroradiol (2009) 30(10):1817–23. 10.3174/ajnr.A1605 19797791 PMC7051282

[13] Goldenberg D Begum S Westra WH Khan Z Sciubba J Pai SI Cystic lymph node metastasis in patients with head and neck cancer: An HPV-associated phenomenon. Head Neck (2008) 30(7):898–903. 10.1002/hed.20796 18383529

[14] Kassirian S Dzioba A Hamel S Patel K Sahovaler A Palma DA Delay in diagnosis of patients with head-and-neck cancer in Canada: Impact of patient and provider delay. Curr Oncol (2020) 27(5):e467–e77. 10.3747/co.27.6547 33173386 PMC7606041

[15] Felippu AW Freire EC Silva Rde A Guimaraes AV Dedivitis RA. Impact of delay in the diagnosis and treatment of head and neck cancer. Braz J Otorhinolaryngol (2016) 82(2):140–3. 10.1016/j.bjorl.2015.10.009 26631328 PMC9449064

[16] Amir Z Kwan SY Landes D Feber T Williams SA. Diagnostic delays in head and neck cancers. Eur J Cancer Care (Engl) (1999) 8(4):198–203. 10.1046/j.1365-2354.1999.00165.x 10889616

[17] Ganesan S Sivagnanganesan S Thulasingam M Karunanithi G Kalaiarasi K Ravichandran S Diagnostic delay for head and neck cancer in South India: A mixed-methods study. Asian Pac J Cancer Prev (2020) 21(6):1673–8. 10.31557/APJCP.2020.21.6.1673 32592363 PMC7568875

[18] Smith MM Abrol A Gardner GM. Assessing delays in laryngeal cancer treatment. Laryngoscope (2016) 126(7):1612–5. 10.1002/lary.25734 26485314

[19] Liao DZ Schlecht NF Rosenblatt G Kinkhabwala CM Leonard JA Ference RS Association of delayed time to treatment initiation with overall survival and recurrence among patients with head and neck squamous cell carcinoma in an underserved urban population. JAMA Otolaryngol Head Neck Surg (2019) 145(11):1001–9. 10.1001/jamaoto.2019.2414 31513264 PMC6743055

[20] Sargeran K Murtomaa H Safavi SM Teronen O. Delayed diagnosis of oral cancer in Iran: Challenge for prevention. Oral Health Prev Dent (2009) 7(1):69–76. 10.3290/j.ohpd.a15270 19408818

